# Reduced platelet hyper-reactivity and platelet-leukocyte aggregation after periodontal therapy

**DOI:** 10.1186/s12959-016-0125-x

**Published:** 2017-02-06

**Authors:** Efthymios Arvanitidis, Sergio Bizzarro, Elena Alvarez Rodriguez, Bruno G. Loos, Elena A. Nicu

**Affiliations:** Department of Periodontology, Academic Centre for Dentistry Amsterdam (ACTA), University of Amsterdam and VU University Amsterdam, Gustav Mahlerlaan 3004, Amsterdam, 1081LA The Netherlands

**Keywords:** Periodontal treatment, Periodontitis, Platelet reactivity, Platelet-monocyte complexes, Platelet-neutrophil Complexes

## Abstract

**Background:**

Platelets from untreated periodontitis patients are hyper-reactive and form more platelet-leukocyte complexes compared to cells from individuals without periodontitis. It is not known whether the improvement of the periodontal condition achievable by therapy has beneficial effects on the platelet function. We aimed to assess the effects of periodontal therapy on platelet reactivity.

**Methods:**

Patients with periodontitis (*n* = 25) but unaffected by any other medical condition or medication were included and donated blood before and after periodontal therapy. Reactivity to ADP or oral bacteria was assessed by flow cytometric analysis of membrane markers (binding of PAC-1, P-selectin, CD63) and platelet-leukocyte complex formation. Reactivity values were expressed as ratio between the stimulated and unstimulated sample. Plasma levels of soluble (s) P-selectin were determined by enzyme-linked immunosorbent assay (ELISA).

**Results:**

Binding of PAC-1, the expression of P-selectin and CD63 in response to the oral bacterium *P. gingivalis* were lower at recall (1.4 ± 1.1, 1.5 ± 1.2, and 1.0 ± 0.1) than at baseline (2.7 ± 4.1, *P* = 0.026, 6.0 ± 12.5, *P* = 0.045, and 2.7 ± 6.7, *P* = 0.042, respectively). Formation of platelet-leukocyte complexes in response to *P. gingivalis* was also reduced at recall compared to baseline (1.2 ± 0.7 vs. 11.4 ± 50.5, *P* = 0.045). sP-selectin levels were significantly increased post-therapy.

**Conclusions:**

In periodontitis patients, the improvement of the periodontal condition is paralleled by a reduction in platelet hyper-reactivity. We suggest that periodontal therapy, as an intervention for improved oral health, can facilitate the management of thrombotic risk, and on the long term can contribute to the prevention of cardiovascular events in patients at risk.

**Trial registration:**

Current Controlled Trials identifier ISRCTN36043780. Retrospectively registered 25 September 2013.

**Electronic supplementary material:**

The online version of this article (doi:10.1186/s12959-016-0125-x) contains supplementary material, which is available to authorized users.

## Background

Periodontitis is a chronic inflammatory disease of the teeth-supporting tissues characterized by progressive loss of attachment, deepening and ulceration of the barrier epithelium [1]. Under these circumstances, bacteremia with oral microorganisms is a common phenomenon during everyday activities, such as chewing and tooth brushing [2]. Oral bacterial species (among them *Porphyromonas gingivalis* and *Streptococcus sanguis)* have been identified in atherosclerotic plaques [3] and are involved in the pathogenesis of infective endocarditis [4]. Mechanisms of action include the capacity of oral bacteria to activate platelets [5, 6] and the formation of platelet-leukocyte complexes, containing activated platelets and leukocytes [7, 8].

Although periodontitis is a common condition (47% of U.S. dentate adults aged 30 years and older [9]) associated with chronic low-grade inflammatory state and atherosclerotic vascular disease [10], it is only sparsely investigated as a potential source of ongoing platelet activation. A previous study from our group has shown that platelets are more activated in periodontitis patients [11]. Besides the more activated state, the platelets and their complexes with leukocytes are hyper-reactive in response to ADP and oral bacteria [12].

It is not known whether periodontal therapy could affect platelet hyper-reactivity thereby facilitating a better management of the cardiovascular risk. We hypothesized that circulating platelets and platelet-leukocyte complexes from peripheral blood of periodontitis patients become less reactive upon stimulation after the provision of non-surgical periodontal therapy. Therefore, the aim of this study was to investigate the effect of non-surgical periodontal therapy on platelet reactivity. To this end, we analyzed platelet membrane-bound activation markers expression and the formation of platelet-leukocyte complexes in response to ADP and oral bacteria (*Aggregatibacter actinomycetemcomitans* [*Aa*], *Porphyromonas gingivalis* [*Pg*], *Tannerella forsythia* [*Tf*], *Streptococcus sanguis* [*Ss*] and *Streptococcus mutans* [*Sm*]).

## Methods

### Chemicals and antibodies

All chemicals were purchased from Sigma Chemical (St Louis, MO, USA). The HEPES buffer solution consisted of 137 mM NaCl, 2.7 mM KCl, 1.0 mM MgCl_2_, 5.6 mM glucose, 20 mM HEPES, 1 mg/mL bovine serum albumin, 3.3 mM NaH_2_PO_4_, pH 7.4. The lysing solution contained 155 mM NH_4_Cl, 10 mM KHCO_3_, 0.1 mM EDTA, pH 7.4 and was stored at 4 °C. The antibodies against surface markers were: CD4-PE, CD14-PE, CD45-APC, CD61-PerCP, CD62P-PE, CD62P-FITC, CD63-PE, CD66b-FITC, PAC-1 FITC, and their isotype control antibodies (all from BD Pharmingen, San Jose, CA, USA). CD61-APC was from Dako (Glostrup, Denmark).

### Bacterial strains and culture conditions

For this experiment, *Aa*, *Pg*, *Tf*, *Ss* and *Sm* were grown as described in a previous study [12]. The bacterial suspensions were washed by centrifugation, reduced to an optical density of 1 at 600 nm in HEPES-buffer and stored in aliquots at −20 °C.

### Patient selection and study protocol

The study population is part of a cohort of moderate-to-severe adult periodontitis patients participating in a clinical trial in the Department of Periodontology at the Academic Centre for Dentistry of Amsterdam (the Netherlands) [13]. Within a period of eighteen months (February 2012-September 2013), all consecutive patients fulfilling the inclusion criteria were included in this study. Patients were recruited if they fulfilled the following inclusion criteria: presence of chronic periodontitis, self–reported good general health and not being aware of any form of diabetes, cardiovascular disease, (auto) immune disease or any other systemic or metabolic disease, and not receiving any medication for hypertension, dyslipidemia of hyperglycemia. Further exclusion criteria were: regular use of medications that could influence platelet function (i.e. NSAIDs, acetylsalicylic acid, dipyridamole, thienopyridines), use of antibiotics in the past 6 months, periodontal treatment in the last 2 years, pregnancy or lactation, presence of implants or orthodontic appliances and presence of <20 natural teeth.

Participants were scheduled for two sessions of non-surgical periodontal therapy (average duration of 5 h) under local anesthesia conducted by two experienced staff oral hygienists. All subjects received a demonstration of basic oral hygiene principles with an electric toothbrush (Philips Sonicare®, Bothell, WA, USA) and were instructed to use interdental means of cleaning followed by rinsing with chlorhexidine 0.12% twice daily. The patients were recalled at six weeks for reinforcement of oral hygiene and localized rescaling of bleeding pockets. Patients were subsequently enrolled in a 3-monthly maintenance program. At baseline and recall (3 months after treatment) blood samples were taken into 0.32% citrate vacuum tubes (BD Vacutainer blood collection tube, Becton Dickinson, Oxford, UK). Citrated plasma was prepared by centrifugation (2000 g, 4 °C, 10 min). Aliquots were stored at −80 °C.

### Platelet reactivity

At baseline and the 3 months-recall (twelve weeks after completion of active periodontal therapy), fasting blood samples for platelet assays were collected between 08:00–10:00 a.m. by venipuncture of the antecubital fossa (0.32% sodium citrate containing blood collection tubes). Blood was kept at room temperature and processed within 30 min of collection.

Aliquots of whole blood (10 μL) were diluted in 30 μL of HEPES-buffer (unstimulated control) or incubated with bacterial suspensions of Aa, Pg, Tf, Ss and Sm. Adenosine diphosphate (ADP, 10 μM) was used as a positive control [14]. The reaction vials contained APC-labeled anti-CD61, FITC-labeled PAC-1 plus PE-labeled anti-CD62P or APC-labeled anti-CD61 plus PE-labeled anti-CD63 (4 μg/mL, final concentration) [15]. To set fluorescence thresholds, 4 μg/mL PE-IgG_1_ and 4 μg/mL FITC-IgM isotype control antibodies were used. The mixes were allowed to incubate for 30 min at room temperature in the dark. The samples were fixed by adding HEPES-buffer containing 0.3% paraformaldehyde (PFA, 2.5 mL). Whole blood flow cytometry was conducted on Accuri™ flow cytometer employing C6 software (Becton Dickinson, Michigan, USA). Forward (size) and side (granularity) scatter were set at logarithmic gain and the geometric mean fluorescent intensity (MFI) was recorded. Platelets were identified on basis of a characteristic forward and side scatter and specific binding of CD61. Within the platelet gate, fluorescence was employed to distinguish between PAC-1, CD62P or CD63 positive cells. Exposure of platelet activation markers was determined on 2500 platelets. The threshold for platelet activation was set at 1% of the appropriate isotype control-antibody.

### Platelet-leukocyte complexes

**Fig. 1:**
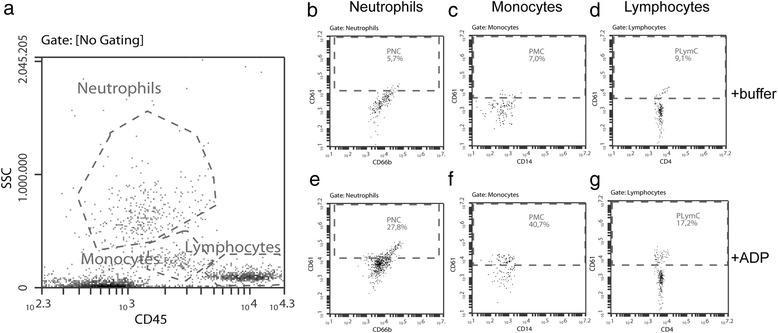
Flow cytometric features of leukocytes, neutrophils, monocytes, and lymphocytes and their complexes with platelets. Panel **a** is showing all events. The Neutrophils gate is characterized by high side scatter and low CD45 expression, the Monocytes gate is characterized by intermediate side scatter and intermediate CD45 expression, and the Lymphocyte gate is characterized by low side scatter and high CD45 expression. All events within the Neutrophils gate in **a** were represented in a CD66b and CD61 plot. Events characterized by high expression of both CD66b and CD61 designate the platelet-neutrophil complexes (PNCs, dotted box in **b**. Similar strategy was employed for the monocytes (using CD14, the platelet-monocyte complexes, PMCs, are in the dotted box in **c** and lymphocytes (using CD4, the platelet-lymphocyte complexes, PLymCs, are in the dotted box in **d**. An example of an unstimulated sample (incubated in HEPES buffer) is shown in **b**, **c**, and **d**, whereas flow cytometric analysis of a stimulated sample (incubated in ADP) is shown in **e**, **f**, and **g**

For the platelet-leukocytes essays, fresh citrated blood (20 μL) was incubated with or without stimulants (see Platelet Reactivity), at room temperature in the dark. Leukocytes were identified using the 90°-light scatter and the expression of CD45 (Fig. 1a). Within each subpopulation gate (neutrophils, monocytes, lymphocytes), the expression of CD61 was determined (Fig.1b-g). The identity of neutrophils, monocytes, and lymphocytes was confirmed by the characteristic expression of CD66b, CD14 and CD4, respectively. The threshold for platelet-leukocyte binding was set at 1% using an isotype control antibody corresponding to the non-specific binding; above this threshold all leukocytes were considered to be CD61 positive reflecting the platelet-leukocyte complexes. After a 15 min incubation, 500 μL of cold (4 °C) lysing solution was added and each sample was placed on ice. Upon lysis, fixation was achieved with the addition of 0.3% PFA solution.

### Soluble P-selectin determination

The concentration of circulating P-selectin in plasma was measured with a commercial enzyme-linked immunosorbent assay (sP-Selectin/CD62p ELISA kit, R&D Systems Inc., Minneapolis, MN, USA). The measurements were performed following the manufacturer’s instructions. For sP-selectin determination, plasma samples were diluted 20 fold. The diluted plasma specimens were added together with an anti-sP-selectin antibody conjugated to peroxidase into a 96-well plate precoated with another anti-sP-selectin monoclonal antibody (MAb) and incubated for 1 h. Substrate was then added, and absorbance values were measured at 450 nm with a plate reader (Synergy HT, BioTek, Winooski, VT, USA). The concentration of each sample was determined by extrapolation from a standard curve estimated from a panel of sP-selectin standards of known concentrations and corrected for the dilution effect of the liquid anticoagulant (citrate). Plasma levels of sP-selectin were expressed as ng/mL. The intra-assay and inter-assay coefficients of variance were 5.2% and 8.7%, respectively.

### Statistical analysis

Our sample size calculation was based on the observed mean ± SD of the fold increase in P-selectin (CD62p) expression on platelets after bacterial stimulation as reported by Nicu et al. [12]. We calculated that we needed to include 29 patients to be able to detect a reduction in platelet reactivity after periodontal therapy with a power of 85% (using a two-sided alpha level of 0.05).

Statistical analysis was performed using SPSS software (v.20.0; IBM, Chicago, Illinois, USA). GraphPad Prism (v. 6.0; GraphPad Software, San Diego, California, USA) was used for the graphic representation of all data. Normality of the data distribution was assessed by Kolmogorov-Smirnov test. When using parametric testing, the data sets showing a non-normal distribution were log-transformed before statistical analysis. The anthropometric, biochemical and clinical characteristics at baseline and recall were compared using the paired *t*-test.

Platelet reactivity was expressed as the ratio between the mean fluorescent intensity (MFI) of the indicated activation marker after stimulation and the MFI of the same marker in the absence of stimulation (cells in HEPES-buffer). The number of CD61-positive (equivalent to platelet-conjugated) leukocytes, neutrophils, monocytes and lymphocytes was recorded as a percentage of the total population of each respectively. Ratios were calculated [% platelet-bound cells after stimulation/% platelet-bound cells in HEPES-buffer] representing the change in number of complexes formed in response to stimulation with oral bacteria or ADP. A general linear model (repeated measures ANOVA using the Huyhn-Feldt correction followed by Bonferroni correction for multiple comparisons), was applied. In this model, comparisons between stimulated (ADP, *Aa*, *Pg*, *Tf*, *Ss*, *Sm*) and unstimulated samples (Hepes) were acquired (*within timepoint* comparisons, at baseline and recall). Secondly, *between timepoints* (baseline and recall) comparisons for each stimulus were assessed by applying the paired *t*-test. *P*-values < 0.05 were considered statistically significant.

*The fold changes over time* in PAC-1, CD62p, CD63, platelet-leukocyte, platelet-neutrophil or platelet-monocyte complexes, LDL levels, systolic blood pressure, waist circumference, number of periodontal pockets deeper than 6 mm were calculated by dividing the value of each parameter at recall by its value at baseline. The *fold changes over time* in PAC-1 (in response to ADP, *Pg*, *Sm*), CD62p (*Pg*), CD63 (*Pg*), platelet-leukocyte (*Pg*), platelet-neutrophil (*Tf*, *Ss*) or platelet-monocyte complexes (*Ss*) were entered as dependent variables, and the amount of smoking (packyears), change in LDL levels, change in systolic blood pressure, change in waist circumference, change in number of periodontal pockets deeper than 6 mm as predictors. Multiple linear regression (Enter method) was used to model the relationship between the calculated platelet-related parameters and these predictors.

## Results

### Patient characteristics and effect of treatment

Within the study period thirty consecutive patients were included in this study; however, due to scheduling conflicts (regarding blood collection and processing), only 25 data sets were available as a complete set (baseline and recall at 12 weeks post-therapy). Demographic and dental characteristics of the participants are described in Table 1. The prevalence of chronic smokers (>10 years) within this group was high (56%). Interestingly, six patients successfully quitted smoking within the duration of the study. Two subjects were classified as obese (BMI > 30 kg/m^2^, waist circumference > 124 cm), while hypercholesterolemia (total cholesterol > 6.2 mmol/L) was noted for three subjects. Using the biochemistry results (summarized in Table 1) we gave the patients a composite score of metabolic syndrome at baseline, according to the presence of central obesity (WC ≥102 cm in men or ≥88 cm in women) together with ≥2 of the following risk determinants: triglycerides ≥1.7 mmol/l, HDL <1.03 mmol/l in males or <1.29 mmol/l in females, blood pressure ≥130/85 mmHg, fasting glucose ≥5.6 mmol/l [16]. Based on this composite score, only 3 out of the 25 participants scored as having metabolic syndrome. LDL levels were lower at recall compared to baseline (*P* = 0.03). Further, seven patients received systemic antimicrobials (375 mg amoxicillin and 250 mg metronidazole, t.i.d, 7 days) as an adjunct to non-surgical periodontal therapy. The leukocyte and platelet counts were comparable before and after periodontal therapy (*P* = 0.852 and 0.570, respectively). As expected, the treatment resulted in significant improvement in all clinical periodontal variables (*P* < 0.001, Table 1). Four teeth were extracted in total, three of them in one individual.

**Table 1:** Summary of the study population characteristics (*n* = 25) at baseline and recall (12 weeks post-therapy)

	Baseline	Recall	*P*-value
Age	44.7 ± 9.3	-	-
Gender (male/female)	11/14	-	-
Ethnicity (Caucasian/non-Caucasian)	21/4	-	-
Smoking (current/former/never)	14/5/6	8/11/6	-
Packyears	16.5 ± 13.8	-	-
BMI (kg/m^2^)	25.0 ± 5.1	25.3 ± 5.2	NS
Waist circumference (cm)	95.6 ± 17.2	94.8 ± 16.9	NS
Blood pressure (mm Hg)			
Systolic	124.8 ± 19.4	127.2 ± 16.1	NS
Diastolic	76.1 ± 13.4	77.2 ± 11.9	NS
Total cholesterol (mmol/L)	5.2 ± 0.8	4.9 ± 0.9	NS
HDL (mmol/L)	1.5 ± 0.4	1.4 ± 0.5	NS
LDL (mmol/L)	3.2 ± 0.8	2.8 ± 0.7	*P* = 0.03
Triglycerides (mmol/L)	1.2 ± 1.0	1.4 ± 1.1	NS
C-reactive protein (mg/L)	2.1 ± 2.8	2.0 ± 3.9	NS
Fibrinogen (g/L)	2.9 ± 0.6	2.8 ± 0.6	NS
Leukocyte counts (× 10^9^/L)	6.7 ± 2.0	6.6 ± 2.2	NS
Neutrophils	3.9 ± 1.6	3.8 ± 1.9	NS
Lymphocyte counts	2.0 ± 0.6	2.2 ± 1.1	NS
Platelet counts (× 10^3^)	250.4 ± 66.1	246.6 ± 56.6	NS
Number of teeth	27.6 ± 2.6	27.4 ± 2.6	NS
#teeth with > 50% bone loss	4.4 ± 3.1	4.1 ± 3.1	NS
Sites with plaque (%)	74.6 ± 20.0	19.0 ± 13.0	*P* < 0.001
Sites with bleeding on probing	65.9 ± 15.0	18.7 ± 9.1	*P* < 0.001
Probing Pocket Depth (mm)	4.0 ± 0.6	2.9 ± 0.4	*P* < 0.001
Pockets ≥ 5 mm (%)	32.4 ± 14.5	11.7 ± 9.0	*P* < 0.001
Clinical Attachment Level (mm)	4.3 ± 0.9	3.6 ± 0.8	*P* < 0.001

Values are presented as means ± standard deviations or number of subjects

*BMI* (body mass index), *HDL* (high density lipoproteins), *LDL* (low density lipoproteins)

### Platelet reactivity

**Fig. 2:**
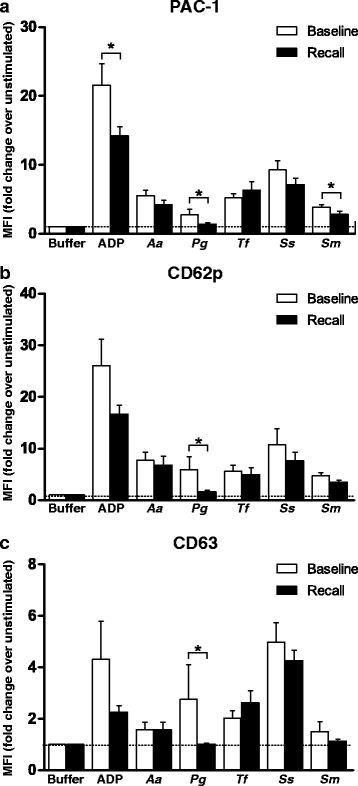
Platelet response to stimulation with ADP or oral bacteria (*Aggregatibacter actinomycetemcomitans* [*Aa*], *Porphyromonas gingivalis* [*Pg*], *Tannerella forsythia* [*Tf*], *Streptococcus sanguis* [*Ss*] and *Streptococcus mutans* [*Sm*]). The mean fluorescence intensity (MFI) of (**a**) PAC-1 binding, (**b**) P-selectin (CD62P) and (**c**) CD63 in response to an agonist was recorded as a measure of reactivity. The data were plotted as fold change in MFI [MFI of individual sample after stimulation/MFI of unstimulated sample in buffer] representing the change in reactivity. Data are presented as means ± standard error of the mean (*N* = 25). Addition of stimuli induced an increase in platelet surface activation markers when analyzed within each timepoint (*P* < 0.001 - repeated measures ANOVA). The comparisons *between* timepoints were analyzed by paired T- test (*P < 0.05, baseline vs. recall)

To determine the reactivity of circulating platelets, P-selectin and CD63 expression, and PAC-1 binding in response to stimulation was assessed (Fig. 2). Stimulation with ADP led to a significant many-fold increase in platelet activation over the unstimulated sample (Fig. 2a, b, c). Similarly, co-incubation of whole blood with oral bacteria induced a smaller but significant amount of platelet activation (overall *P* < 0.001 for the ***within timepoint*** analysis for all three markers). More specifically, *Ss* was the strongest inducer of platelet activation (post hoc *P* < 0.05 for all three markers). The PAC-1 binding was lower at recall than at baseline in response to stimulation with ADP (mean ratio MFI_recall_ = 14.3 vs. mean ratio MFI_baseline_ = 21.6, *P* = 0.042), *Pg* (mean ratio MFI_recall_ = 1.4 vs. mean ratio MFI_baseline_ = 2.7, *P* = 0.026) and *Sm* (mean ratio MFI_recall_ = 2.9 vs. mean ratio MFI_baseline_ = 3.9, *P* = 0.017, Fig. 2a). A non-significant reduction in PAC-1 binding was measured at recall in response to *Aa* and *Ss*. The P-selectin expression in response to *Pg* was lower at recall than baseline (mean ratio MFI_recall_ = 1.6 vs. mean ratio MFI_baseline_ = 6.0, *P* = 0.045, Fig. 2b). Similarly, CD63 expression after *Pg* stimulation was lower at recall compared to baseline (mean ratio MFI_recall_ = 1.0 vs. mean ratio MFI_baseline_ = 2.7, *P* = 0.042, Fig. 2c).

### Formation of platelet-leukocyte complexes

**Fig. 3:**
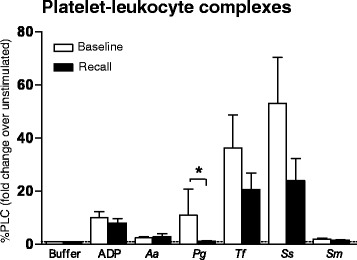
Formation of platelet-leukocyte complexes in response to stimulation with ADP or oral bacteria (*Aggregatibacter actinomycetemcomitans* [*Aa*], *Porphyromonas gingivalis* [*Pg*], *Tannerella forsythia* [*Tf*], *Streptococcus sanguis* [*Ss*] and *Streptococcus mutans* [*Sm*]). The number of CD61-positive (equivalent to platelet-conjugated) leukocytes (PLC) was recorded as a percentage of the total population. Data were plotted as fold change [% platelet-bound cells after stimulation/% platelet-bound cells in unstimulated sample in buffer] representing the change in number of complexes formed in response to stimulation with ADP or oral bacteria. Data are presented as means ± standard error of the mean (*N* = 25). The addition of stimuli induced formation of complexes when analyzed within each timepoint (*P* < 0.001 in repeated measures ANOVA). The comparisons *between* timepoints were analyzed by paired T- test (*P < 0.05 baseline vs. recall)

Incubation of fresh platelets with ADP or oral bacteria resulted in formation of platelet-leukocyte complexes (PLC) both at baseline and recall (overall *P* < 0.001 for the ***within*** *timepoints* analysis, Fig. 3). Specifically, formation of platelet-neutrophil (PNC) and platelet-monocyte complexes (PMC) was significantly induced by stimulation (Table 2). The strongest inducers of PLC, PNC or PMC were ADP, *Tf* and *Ss* (*post-hoc P* < 0.05). Formation of platelets-lymphocyte complexes (PLymC) appeared less influenced either by ADP or oral bacteria (Table 2, *P* > 0.05).

**Table 2:** Summary of platelet-neutrophil, platelet-monocyte and platelet-lymphocyte complexes formation in response to stimulation

Platelet-neutrophil complexes (Fold change over unstimulated)				Platelet-monocyte complexes (Fold change over unstimulated)				Platelet-lymphocyte complexes (Fold change over unstimulated)			
	Baseline	Recall	*P*-value		Baseline	Recall	*P*-value		Baseline	Recall	*P*-value
**+**ADP	6.9 ± 7.9	8.6 ± 19.3	0.468	**+**ADP	13.0 ± 19.4	8.4 ± 16.8	0.339	**+**ADP	4.7 ± 8.1	7.1 ± 10.5	0.072
*+Aa*	2.1 ± 1.8	6.1 ± 17.5	0.766	+*Aa*	2.7 ± 2.5	1.9 ± 1.3	0.265	+*Aa*	7.7 ± 19.2	3.9 ± 8.2	0.483
+*Pg*	9.4 ± 40.1	2.4 ± 5.6	0.462	+*Pg*	2.1 ± 3.2	1.2 ± 1.0	0.100	+*Pg*	1.8 ± 2.1	2.0 ± 3.0	0.535
+*Tf*	32.9 ± 56.2	14.9 ± 30.5	**0.028**	+*Tf*	8.7 ± 8.3	7.7 ± 19.6	0.072	+*Tf*	11.5 ± 23.2	9.3 ± 12.1	0.431
+*Ss*	51.3 ± 93.4	15.4 ± 31.9	**0.003**	+*Ss*	13.2 ± 20.1	7.0 ± 25.1	**0.004**	+*Ss*	4.1 ± 10.6	3.9 ± 5.9	0.529
+*Sm*	1.4 ± 1.4	2.7 ± 7.8	0.906	+*Sm*	2.2 ± 2.0	1.2 ± 1.2	**0.046**	+*Sm*	1.4 ± 0.8	2.7 ± 3.8	0.087

Values are presented as means ± standard deviations. The platelet-neutrophil complexes, platelet-monocyte complexes, platelet-lymphocyte complexes were calculated as percentages platelet-positive neutrophils, monocytes or lymphocytes from the total number of neutrophils, monocytes or lymphocytes, respectively. The fold change over unstimulated values represent the ratios between the measured values after stimulation with ADP, Aa, Pg, Tf, Ss or Sm and in the absence of stimulation (cells in buffer). *P*-values were obtained by paired *T*-test and the significant values are given in bold

*ADP* (adenosine diphosphate), *Aa* (Aggregatibacter actinomycetemcomitans), *Pg* (Porphyromonas gingivalis), *Tf* (Tannerella forsythia), *Ss* (Streptococcus sanguis), *Sm* (Streptococcus mutans)

Less PLC formation could be observed at recall compared to baseline in response to *Pg* (mean ratio %PLC_recall_ = 1.2 vs. mean ratio %PLC_baseline_ = 11.4, *P* = 0.045, Fig. 3). Furthermore, less PNC were formed at recall compared to baseline in response to *Tf* (*P* = 0.045) and *Ss* (*P* = 0.009, Table 2). Similarly, less PMCs were formed at recall in response to *Tf* (*P* = 0.065), *Ss* (*P* = 0.004), and *Sm* (*P* = 0.093). PLymC formation was unaffected by time or bacterial stimulation (*P* > 0.05, Table 2).

The intrinsic platelet activation status at recall compared to baseline is presented in Additional file 1, in which raw data of the unstimulated condition (samples incubated in HEPES buffer) are summarized. None of the tested parameters were significantly different between baseline and recall. We noted a trend towards higher PAC-1 binding 3 months post-treatment (*P* = 0.061) and higher percentage of platelet-neutrophils complexes (*P* = 0.077), which however did not reach statistical significance.

### Soluble P-selectin concentration

**Fig. 4:**
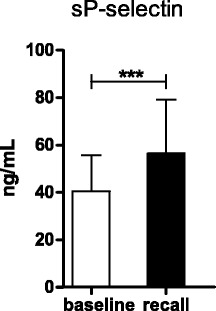
Concentration of sP-selectin (*N* = 24) in citrate plasma at baseline (white bars) and recall (black bars). Data are presented as means ± standard deviation. *P*-value calculated by paired t- test (***P = 0.0008)

At baseline, sP-selectin values ranged between 15.8 ng/mL and 72.8 ng/mL (mean ± standard deviation 40.5 ± 15.1 ng/mL, Fig. 4). The plasma levels of sP-selectin at recall (range 21.1 - 123.8 ng/mL, mean ± SD: 56.4 ± 22.8 ng/mL) were significantly increased compared to baseline (*P* = 0.0008). The plasma concentration of sP-selectin was not correlated with the platelet expression of P-selectin (CD62p expression in unstimulated samples) at baseline (R = −0.37, *P* = 0.863) or recall (*R* = 0.006, *P* = 0.979).

### Linear regression models

Linear regression analyses were run for all the platelet parameters showing a significant decrease post-treatment (PAC-1 binding in response to ADP, *Pg*, and *Sm*, CD62 response to *Pg*, CD63 response to *Pg*, PLC formation in response to *Pg*, PNC formation in response to *Tf*, PNC formation in response to *Ss*, PMC formation in response to *Ss*). The regression models included the following variables as predictors: smoking amounts, LDL (ratio recall/baseline), waist circumference (ratio recall/baseline), systolic blood pressure (ratio recall/baseline) and the clinical response to periodontal treatment (ratio between the number of periodontal pockets deeper than 6 mm at recall/baseline). The results of these regression models are presented in Additional file 2. The final regression equation for the PAC-1 binding in response to ADP yielded R^2^: 0.584, *F* = 3.083; *P* = 0.05. From this regression model it became apparent that the clinical response to periodontal treatment positively predicted the improvement in PAC-1 binding in response to ADP (*B* = 3.26, *t* = 2.76, *P* = 0.02) and *Sm* (*B* = 2.41, *t* = 2.29, *P* = 0.04). The positive relation between PAC-1 binding and response to treatment translates into: the reduction in the number of periodontal pockets deeper than 6 mm after treatment is positively correlated with the reduction in PAC-1 binding in response to ADP or *Sm*.

None of the other variables included in the models predicted the change in platelet parameters (*P* > 0.05).

## Discussion

Many inflammatory states, e.g. systemic lupus erythematosus, pneumonia, or inflammatory bowel disease feature increased platelet reactivity, and potentially hypercoagulability with an increased risk for cardiovascular events [17–20]. Periodontitis is a condition characterized by low-grade inflammatory and infectious burden with systemic distribution of pro-inflammatory cytokines or bacterial products. We have previously documented hyper-reactivity of platelets in response to ADP and oral bacteria in untreated periodontitis patients as compared to matched controls [12]. In the current study we show that the improvement in the periodontal condition after non-surgical periodontal therapy is accompanied by a significant reduction in platelet reactivity, as reflected by lower PAC-1 binding, lower P-selectin expression and lower induction of platelet-leukocyte complexes in response to an agonist.

The circulation of leukocytes across the vascular endothelium is required for immune surveillance and for leukocyte recruitment at inflammatory sites. Platelet binding alters the adhesive and migratory phenotype of leukocytes, with platelet-monocyte and platelet-neutrophil complexes being more adhesive, thus resulting in enhanced transmigration through vascular endothelium [21]. Conversely, when the periodontal inflammatory lesions heal after periodontal therapy, the need for recruitment of inflammatory cells will decrease. This could explain our finding of reduced propensity to form platelet-leukocyte complexes after periodontal treatment. A positive side effect of this therapy could result in a reduced participation of platelets and leukocytes to the progression of atherothrombosis [22–24], potentially benefitting the patients at cardiovascular risk.

It has been reported in (systematic) reviews that successful treatment of periodontitis is accompanied by a decrease in systemic markers of inflammation such as CRP or leukocyte counts [25, 26]. In our study the CRP levels and leukocyte counts did not change after periodontal therapy. However, this finding must be interpreted in the context of the strict exclusion criteria we have applied. In this work, patients with any known co-morbidities were excluded. Although inclusion in the study was based on the self-reported health status, which might underestimate the prevalence of a medical condition when compared to diagnosis based on laboratory data [17], we have strong indications that the large majority of our participants were healthy, as only 3 out of 25 fulfilled the criteria for metabolic syndrome [16]. In the most recent meta-analysis of the effects of periodontal treatment on CRP levels, Teeuw and coworkers analyzed and presented separately the results from subjects with periodontitis, but otherwise healthy (without co-morbidities) and those from subjects with co-morbidities. From their meta-analysis it was concluded that it is the patients with co-morbidities that benefit the most from periodontal therapy, showing consistent reduction in CRP levels after treatment, whereas in the group without co-morbidities the change in CRP levels over time after periodontal treatment was not different than in the untreated control group [27]. An explanation for this finding might be the low baseline CRP values in most of the participants in our study (only 3 patients had CRP above the high risk AHA threshold of 3.0 mg/L). Ridker and coworkers reported a significant reduction in the risk for myocardial infarction after aspirin administration in the highest quartile of baseline CRP levels, but not in the lowest quartile [28]. The authors concluded that a certain inflammatory threshold might be necessary before anti-infective/inflammatory treatments can deliver measurable effects, and we suggest that this is the mechanism behind the non-significant change in inflammatory markers in our study population after periodontal treatment.

Interestingly, in the current study, the LDL levels were reduced after periodontal therapy. Our results are in line with previous reports of improved serum lipid levels post periodontal therapy in patients with periodontitis, but otherwise healthy, in the absence of any adjunctive lipid-lowering intervention (dietary advice or pharmacological cholesterol reduction medication) [29, 30]. Similar to the effects that periodontal therapy has on CRP levels, the lipid profiles show greater improvement in periodontitis patients with co-morbidities, e.g. hyperlipidemia, diabetes [31–33].

P-selectin is expressed on the cell surface of platelets and endothelial cells after activation [34], and mediates leukocyte adhesion to these activated cell types. We found a statistically significant, albeit modest, increase 3 months after periodontal therapy of sP-selectin levels. A similar trend post-treatment was observed by Marcaccini et al. [35]. Interestingly, also the unstimulated platelet activation values, representing intrinsic activity, showed a trend of slight increase (Additional file 1). It would be interesting to see in future studies longer term results (more than 6 months) for these parameters. Activated platelets rapidly shed their membrane-bound P-selectin, which subsequently can be found as circulating sP-selectin [36], while platelets remain functional and responsive to stimulation. Possibly, this latter phenomenon explains why in periodontitis patients we found increased sP-selectin post-therapy, while their platelet reactivity was reduced. However, the increased sP-selectin post-therapy might be a marker of early endothelial function recovery. Previous observations have shown that in vivo, the adhesiveness of the endothelium is controlled by shedding of P-selectin [37]. Moreover, the shed soluble P-selectin is likely to inhibit additional leukocyte adhesion and may have a “calming” effect on the recruited neutrophils [38], which again may be part of the healing processes initiated post-therapy. Indeed, periodontal therapy has been shown to improve endothelial dysfunction [39], and our results provide insights into the cellular players involved.

Although slightly increased post-treatment, the sP-selectin levels in our periodontitis patients were lower than previously reported for both untreated periodontitis or healthy (periodontally unaffected) controls [11, 35]. We are cautious in interpreting these results, mainly because in the current study we measured sP-selectin in citrate plasma, and not in EDTA-plasma, like in the studies by Marcaccini et al. and Papapanagiotou et al. It has been shown that sP-selectin is lower in citrate than in EDTA [40], rendering comparisons between studies employing different anticoagulants highly hazardous.

The most widely used methods for the analysis of platelet function in vitro include global tests for whole blood such as the Platelet Function Analyzer 100 (PFA-100®), Plateletworks®, VerifyNow® or thromboelastography and specific tests such as the light transmission aggregometry (LTA), the flow cytometry-based tests or the ELISA-based tests [41]. Global tests are useful in monitoring the individual on-treatment platelet reactivity in clinical settings [42], however, they lack sensitivity and specificity due to interplay with multiple non-platelet related factors, e.g. plasma-derived adhesion and (anti)-coagulation factors, red blood cells and leukocytes [41]. LTA, on the other hand, enables the analysis of distinct activation mechanisms on isolated platelets (platelet-rich plasma or plasma-depleted platelet preparations) and represents the “gold standard” of platelet function analysis in vitro. However, LTA lacks the attributes needed for a point-of-care test: it requires sample preparation, is time consuming, operator-sensitive and has limited recommendation for monitoring anti-platelet therapy responses [41].

The methods employed in the current study, i.e. the flow cytometric evaluation of platelet surface markers and the determination of soluble P-selectin concentration in plasma by ELISA are both of a static nature. Ideally, platelet functions should be assessed under flow conditions, since some biologically relevant interactions such as that of GP1bα and VWF are relevant under shear stress. The hospital based analyzers such as the PFA-100® and VerifyNow® incorporate some aspect of shear stress, but are limited by the incomplete analysis of hemostasis they generate, the relative big volume of donor blood required and the low throughput [43]. Promising results have been obtained with microfluidic devices demonstrating the ability to obtain a large number of data points per single patient sample using small blood volumes and high throughput approach [44]. Although none of these flow-based assays were available for the current work, a future study should address the effect of periodontal therapy on platelet functions under flow conditions.

One limitation of the current study is the absence of an untreated (control) group. This would consist of periodontitis patients left untreated for the whole duration of the study. However, the inclusion of such a group is unethical, and would be met with strong opposition by the University Ethical Board. Furthermore, such a control group has been shown to be susceptible to a high dropout rate by patients who seek periodontal therapy elsewhere [45]. Nevertheless, on the basis of the current study it is not possible to establish whether the improvement in platelet reactivity is solely related to the professionally-applied periodontal therapy. It cannot be excluded that some patients have changed their lifestyle after receiving information about the negative effects of smoking, overweight, lack of physical activity or unhealthy diet. The study protocol did not include the assessment of changes in physical activity or diet, which might have been of influence on the measured platelet functions. The LDL levels were lower post-treatment and, by the time of the recall (3 months post-periodontal therapy), six out of the 14 smokers in the current study had quitted smoking. As both LDL and smoking could influence platelet function [46–48], we sought of estimating their effect on the measured platelet parameters by regression analysis. When exploring the predictive value of smoking or the changes in LDL, systolic blood pressure, waist circumference for the measured platelet parameters, no significant effect of these variables was found. However, the study cohort is rather limited, so this conclusion might be underpowered.

In conclusion, our results support the notion of a longitudinal beneficial effect of periodontitis treatment on platelet function. Our results are a promising step towards an increased awareness of the medical community for periodontal therapy as a non-pharmacological intervention for improving platelet function, and given the high prevalence of periodontitis, could potentially benefit a large proportion of the adult population.

## Conclusion

In conclusion, we suggest that periodontal therapy as an intervention for improved oral health is one of the emerging factors, in addition to lifestyle changes (nutrition, physical activity and psychological wellbeing) contributing to a reduction of the intrinsic platelet hyper-reactivity, which on the long term can have substantial benefits in preventing major cardiovascular events.

## Additional files

Additional file 1: Raw values of platelet parameters in unstimulated samples (incubation in HEPES buffer) at baseline and recall (3 months post-therapy). (DOCX 15 kb)
Additional file 2: Predictive factors of changes after treatment in platelet-related parameters according to multiple linear regression analysis. (DOCX 20 kb)
