# Supplementary material for: Reduced platelet hyper-reactivity and platelet-leukocyte aggregation after periodontal therapy
Source: Thromb J. 2017 Feb 6;15:5. doi: 10.1186/s12959-016-0125-x (PMC5292810; doi:10.1186/s12959-016-0125-x)
Supplement: Additional file 1: — Raw values of platelet parameters in unstimulated samples (incubation in HEPES buffer) at baseline and recall (3 months post-therapy). (DOCX 15 kb) [file 12959_2016_125_MOESM1_ESM.docx]

**Additional file 1.** Raw values of platelet parameters in unstimulated samples (incubation in HEPES buffer) at baseline and recall (3 months post-therapy).

|  | **Baseline** | **Recall** | ***P*-value** |
| --- | --- | --- | --- |
| PAC-1 binding (MFI) | 411.9 ± 227.4 | 586.7± 609.0 | 0.061 |
| CD62P (MFI) | 403.9 ± 267.8 | 446.7± 438.2 | 0.585 |
| CD63 (MFI) | 412.6 ± 825.7 | 195.7± 46.6 | 0.203 |
| PLC (%) | 3.6 ± 0.8 | 5.5 ± 1.6 | 0.315 |
| PNC (%) | 10.3 ± 11.9 | 16.8 ± 13.5 | 0.077 |
| PMC (%) | 5.8 ± 1.5 | 7.0 ± 1.9 | 0.597 |
| PLymC (%) | 0.3 ± 0.1 | 0.8 ± 0.4 | 0.187 |

Values are presented as means ± standard deviations. PAC-1 binding, CD62P and CD63 are expressed as MFI (mean fluorescence intensity). PLC (platelet-leukocyte complexes), PNC (platelet-neutrophil complexes), PMC (platelet-monocyte complexes), PLymC (platelet-lymphocyte complexes) are expressed as percentages (%) from the total. *P*-values were obtained by paired T-test.
