# Supplementary material for: Reduced platelet hyper-reactivity and platelet-leukocyte aggregation after periodontal therapy
Source: Thromb J. 2017 Feb 6;15:5. doi: 10.1186/s12959-016-0125-x (PMC5292810; doi:10.1186/s12959-016-0125-x)
Supplement: Additional file 2: — Predictive factors of changes after treatment in platelet-related parameters according to multiple linear regression analysis. (DOCX 20 kb) [file 12959_2016_125_MOESM2_ESM.docx]

**Additional file 2**. Predictive factors of changes after treatment in platelet-related parameters according to multiple linear regression analysis

|  | **PAC-1 binding (response to ADP)** | | | | | **PAC-1 binding (response to *Pg*)** | | | | | **PAC-1 binding(response to *Sm*)** | | | | |
| --- | --- | --- | --- | --- | --- | --- | --- | --- | --- | --- | --- | --- | --- | --- | --- |
| **Independent variables** | B | Std. error | t | *P*-value | 95% CI for B | B | Std. error | t | *P*-value | 95% CI for B | B | Std. error | t | *P*-value | 95% CI for B |
| Smoking (pack years) | 0.01 | 0.01 | 1.34 | 0.21 | -0.01 to 0.04 | 0.01 | 0.01 | 1.08 | 0.30 | -0.01 to 0.02 | -0.01 | -0.01 | -0.94 | 0.37 | -0.03 to 0.01 |
| LDL | -1.62 | 0.88 | -1.85 | 0.09 | -3.55 to 0.31 | 0.82 | 0.46 | 1.79 | 0.10 | -0.19 to 1.83 | -0.61 | 0.78 | -0.78 | 0.45 | -2.33 to 1.11 |
| WC | -0.24 | 1.89 | -0.13 | 0.90 | -4.39 to 3.92 | -1.95 | 0.99 | -1.98 | 0.07 | -4.13 to 0.22 | 1.40 | 1.68 | 0.83 | 0.42 | -2.30 to 5.11 |
| SBP | 2.93 | 3.42 | 0.86 | 0.41 | -4.59 to 10.45 | 0.19 | 1.79 | 0.11 | 0.92 | -3.75 to 4.13 | -1.16 | 3.05 | -0.38 | 0.71 | -7.86 to 5.55 |
| Response to treatment | 3.26 | 1.18 | 2.76 | **0.02** | 0.66 to 5.86 | -0.60 | 0.62 | -0.97 | 0.35 | -1.96 to 0.76 | 2.41 | 1.05 | 2.29 | **0.04** | 0.09 to 4.72 |
|  | R^2^: 0.584; F=3.083; ***P*=0.05** | | | | | R^2^: 0.438; F=1.717; *P*=0.21 | | | | | R^2^: 0.405; F=1.498; *P*=0.27 | | | | |

|  | **CD62p (response to *Pg*)** | | | | | **CD63 (response to *Pg*)** | | | | | **PLC (response to *Pg*)** | | | | |
| --- | --- | --- | --- | --- | --- | --- | --- | --- | --- | --- | --- | --- | --- | --- | --- |
| **Independent variables** | B | Std. error | t | *P*-value | 95% CI for B | B | Std. error | t | *P*-value | 95% CI for B | B | Std. error | t | *P*-value | 95% CI for B |
| Smoking (pack years) | 0.02 | 0.02 | 1.30 | 0.22 | -0.02 to 0.06 | -1.01 | 0.67 | -1.52 | 0.88 | -0.98 to 9.38 | 0.01 | 0.02 | 0.42 | 0.68 | -0.03 to 0.04 |
| LDL | -0.09 | 1.41 | -0.06 | 0.95 | -3.19 to 3.02 | 0.39 | 0.50 | 0.78 | 0.45 | -0.71 to 1.48 | 1.07 | 1.25 | 0.86 | 0.41 | -1.72 to 3.87 |
| WC | 3.49 | 3.04 | 1.15 | 0.27 | -3.20 to 10.18 | -0.45 | 1.07 | -0.42 | 0.68 | -2.81 to 1.90 | -0.86 | 2.68 | -0.32 | 0.75 | -6.84 to 5.11 |
| SBP | -4.48 | 5.50 | -0.82 | 0.43 | -16.59 to 7.63 | -3.16 | 1.94 | -1.63 | 0.13 | -7.42 to 1.10 | -1.53 | 4.83 | -0.32 | 0.76 | -12.29 to 9.24 |
| Response to treatment | -1.63 | 1.90 | -0.86 | 0.41 | -5.82 to 2.55 | -1.01 | 0.67 | -1.52 | 0.16 | -2.49 to 0.46 | 0.35 | 1.71 | 0.20 | 0.84 | -3.46 to 4.16 |
|  | R^2^: 0.292; F=0.907; *P*=0.51 | | | | | R^2^: 0.396; F=1.445; *P*=0.28 | | | | | R^2^: 0.156; F=0.370; *P*=0.86 | | | | |

|  | **PNC (response to *Tf*)** | | | | | **PNC (response to *Ss*)** | | | | | **PMC (response to *Ss*)** | | | | |
| --- | --- | --- | --- | --- | --- | --- | --- | --- | --- | --- | --- | --- | --- | --- | --- |
| **Independent variables** | B | Std. error | t | *P*-value | 95% CI for B | B | Std. error | t | *P*-value | 95% CI for B | B | Std. error | t | *P*-value | 95% CI for B |
| Smoking (pack years) | 0.02 | 0.04 | 0.43 | 0.67 | -0.06 to 0.09 | 0.03 | 0.03 | 1.09 | 0.30 | -0.03 to 0.09 | -1.97 | 1.79 | -1.10 | 0.30 | -0.03 to 0.04 |
| LDL | 6.64 | 2.95 | 2.25 | 0.06 | 0.14 to 13.13 | 3.80 | 2.36 | 1.61 | 0.14 | -1.39 to 8.99 | -198.97 | 151.65 | -1.31 | 0.22 | -532.75 to 134.82 |
| WC | 0.37 | 6.35 | 0.06 | 0.95 | -13.61 to 14.35 | -1.46 | 5.08 | -0.29 | 0.78 | -12.64 to 9.71 | 174.52 | 326.42 | 0.54 | 0.60 | -6.84 to 5.11 |
| SBP | 20.03 | 11.50 | 1.74 | 0.11 | -5.28 to 45.34 | 14.18 | 9.19 | 1.54 | 0.15 | -6.05 to 34.41 | -1155.04 | 591.01 | -1.95 | 0.08 | -2455.84 to 145.76 |
| Response to treatment | -0.03 | 3.97 | -0.01 | 0.99 | -8.77 to 8.71 | -2.09 | 3.17 | -0.66 | 0.52 | -9.08 to 4.89 | -202.12 | 204.15 | -0.99 | 0.34 | -651.45 to 247.20 |
|  | R^2^: 0.365; F=1.264; *P*=0.345 | | | | | R^2^: 0.338; F=1.124; *P*=0.40 | | | | | R^2^: 0.365; F=1.265; *P*=0.34 | | | | |

The regression models included the following variables as predictors: smoking amounts, LDL, waist circumference (WC), systolic blood pressure (SBP) and the clinical response to periodontal treatment. PLC (platelet-leukocyte complexes), PNC (platelet-neutrophil complexes), PMC (platelet-monocyte complexes). ADP (adenosine diphosphate), *Pg* (*Porphyromonas gingivalis*), *Tf* (*Tannerella forsythia)*, *Ss* (*Streptococcus sanguis*) and *Sm* (*Streptococcus mutans*)*.*
